# Impact of pH on the Stability and the Cross-Reactivity of Ochratoxin A and Citrinin

**DOI:** 10.3390/toxins5122324

**Published:** 2013-11-28

**Authors:** Ingrid Bazin, Virginie Faucet-Marquis, Marie-Carmen Monje, Micheline El Khoury, Jean-Louis Marty, Annie Pfohl-Leszkowicz

**Affiliations:** 1 Ecole des mines d’Ales, 6 av de Clavieres, 30100 Ales Cedex, France; E-Mail: micheline.el.khoury@gmail.com; 2 Laboratory Chemical Engineering, Department Bioprocess & Microbial System, University of Toulouse, UMR CNRS/INPT/UPS 5503, 1 Avenue Agrobiopole, 31320 Auzeville-Tolosane, France; E-Mails: virginie.marquis@anabiotox.fr (V.F.-M.); monje@ensat.fr (M.-C.M.); 3 Anabiotox 16 allée Montcalm, 31500 Ramonville, France; 4 Laboratory IMAGES, University of Perpignan, 52 Avenue Paul Alduy, 66860 Perpignan Cedex, France; E-Mail: jlmarty@univ-perp.fr

**Keywords:** mycotoxin, ochratoxin, citrinin, analysis, immunoaffinity, wheat, wine

## Abstract

Mycotoxins are secondary metabolites produced by several fungi contaminating crops. In several countries, the maximum permitted levels of mycotoxins are found in foodstuffs and feedstuffs. The common strategy of mycotoxin analysis involves extraction, clean-up and quantification by chromatography. In this paper, we analyzed the reasons of underestimation of ochratoxin A (OTA) content in wine, and overestimation of OTA in wheat, depending on the pH of the clean-up step and the simultaneous presence of citrinin (CIT). We demonstrated that the increase of pH by adding polyethylene glycol (PEG) to wine led to an underestimation of OTA by conversion of OTA into open ring ochratoxin A OP-OA. In comparing three methods of extraction and clean-up for the determination of OTA and CIT in wheat—(i) an inter-laboratory validated method for OTA in cereals using immunoaffinity column clean-up (IAC) and extraction by acetonitrile/water; (ii) a validated method using IAC and extraction with 1% bicarbonate Na; and (iii) an in-house validated method based on acid liquid/liquid extraction—we observed an overestimation of OTA after immunoaffinity clean-up when CIT is also present in the sample, whereas an underestimation was observed when OTA was alone. Under neutral and alkaline conditions, CIT was partially recognized by OTA antibodies.

## 1. Introduction

**Figure 1:**
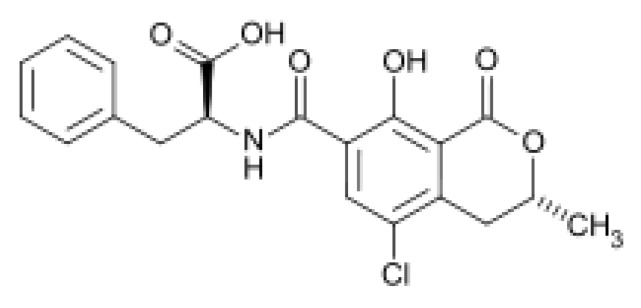
Chemical structure of ochratoxin A (OTA).

*Aspergillus*, *Penicillium* and *Fusarium* are fungi often found in crops. After a period of balanced growth followed by stress conditions, fungi produce a large variety of toxic secondary metabolites called mycotoxins [1]. These compounds have several chemical structures. *Aspergillus ochraceus* and *Penicillium citrinum* are the main producers of ochratoxin A (OTA) and citrinin (CIT), respectively. Ochratoxin A (OTA), 7-(l-β-phenylalanylcarbonyl)-carboxyl-5-chloro-8-hydroxy-3,4-dihydro-3*R*-methylisocumarin (Figure 1), is detected in many stored and dry foodstuffs [2], such as corn, wheat, oats, beans, nuts, peanuts, rice, barley, sorghum, cotton seed, coffee beans, cocoa and spices [3,4,5,6]. OTA is found in different animal tissues [7], as well as in human blood and breast milk [8]. This mycotoxin is a powerful nephrotoxin, teratogen, immunosuppressive agent [9,10]. The International Agency for Research on Cancer (IARC) classified OTA in 2B Group (possibly carcinogenic to human). The European Community placed Maximum Residues Limits (MRL) of OTA on several foodstuffs [6,11]. For example, the limit of OTA in wine is 2 µg/ kg, or 5 µg/kg in unprocessed cereal. 

**Figure 2:**
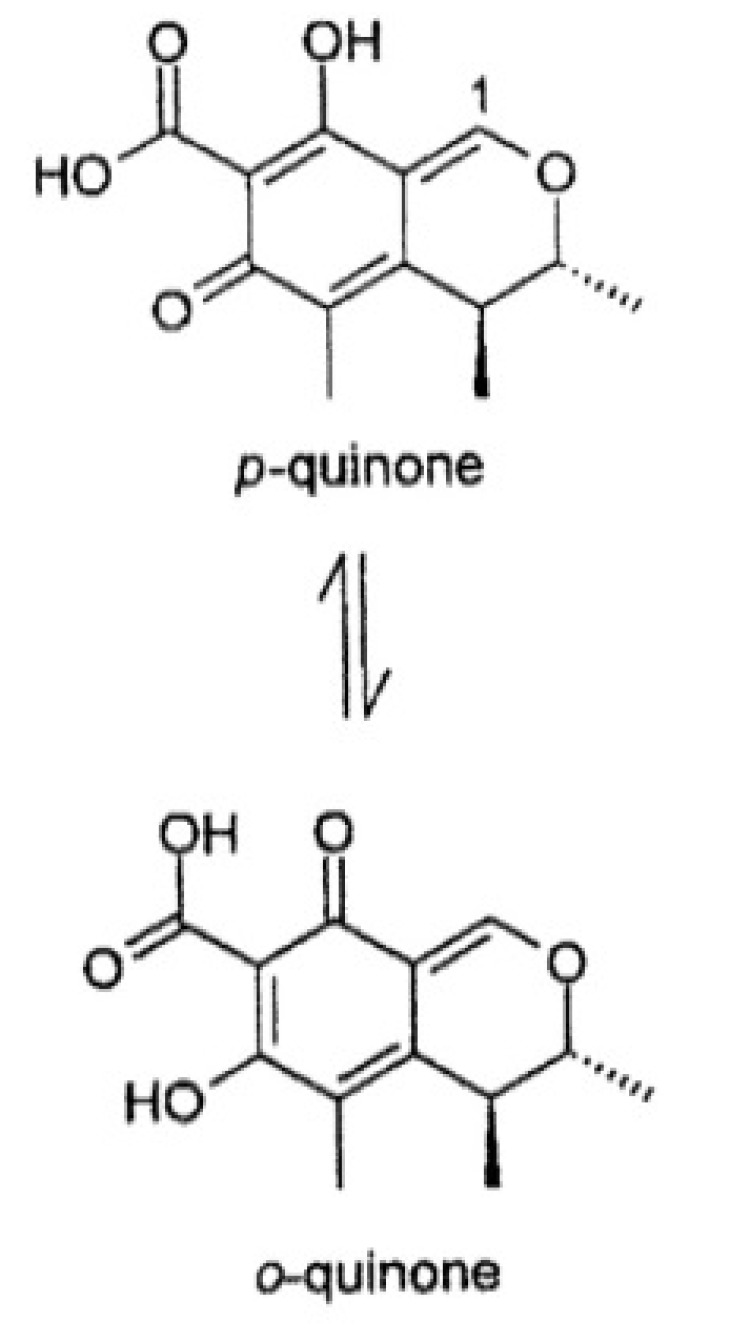
Chemical structure of citrinin, existing in both forms.

Citrinin (CIT; 3*R*,4*S*)-8-hydroxy-3,4,5-trimethyl-6-oxo-4,6-dihydro-3*H*-isochromene-7-carboxylic acid; Figure 2) is a fungal metabolite which was isolated for the first time from *Penicillium citrinum* [12]. The main species of fungi producing CIT belong to the genera *Aspergillus* and *Penicillium*. CIT is nephrotoxic [13] and is involved with OTA as a potential agent of Balkan endemic nephropathy (BEN) [14,15,16]. CIT is genotoxic [17,18,19,20]. It enhances OTA renal toxicity in pigs [13] and rodent renal cancer [9].

As some species of *Penicillium* (such as *P. verrucosum* or *P citrinum*) produce both OTA and CIT [21,22], these two mycotoxins can be found simultaneously in cereals. Co-contamination by CIT-OTA was observed in samples of food such as rice [3,23], olives [24,25]*,* wheat, [4,26,27] and whole meal [15]. 

Co-contamination of mycotoxins still poses a risk due to the possible synergy and their additive effects [15,20,28]. The development of methodologies allowing simultaneous extraction of these mycotoxins in different matrices has been encouraged [29]. In recent years, analytical methods for extraction and analysis of these two mycotoxins have been improved [30,31]. Extractions in liquid phase were largely studied for ochratoxin A [32], and most of the validated methods were based on the extraction of ochratoxin A and citrinin through the solubility of these compounds in organic solvents or alkaline solutions [25,33]. The most widely used technique is HPLC with fluorescence detection. Different excitation and emission fluorescence parameters (OTA 335 and 465 nm; CIT 331 and 500 nm) were used to achieve the optimal conditions of detection for each toxin [4]. However, for this analytical method, an efficient sample extract clean-up is mandatory to reach low detection limits and to protect the HPLC column. The most frequent clean-up method involves solid-phase extraction (SPE) of sample by using an immunoaffinity column [29,34]. This kind of SPE greatly facilitates the clean-up stage, usually providing high purity extracts that can be directly injected in the HPLC column [35,36], and are commonly used for the determination of mycotoxins in different matrices with adequate analytical efficiency. However, their disadvantages are that specific antibodies should exist for each mycotoxin. Until now, no IAC for the determination of CIT is available. A versatile method suitable for raw cereals and cereal products using partition was developed by Molinié *et al.* [4].

The development of new extraction and analysis techniques had been reported. The QuEChERS (Quick, Easy, Cheap, Effective, Rugged and Safe) [37,38,39] method is a procedure that was adopted for the extraction of mycotoxin due to its quickness and low cost. In recent years, there has been a tremendous increase in reports on the determination of OTA and/or citrinin immunosensors based on different platforms. Rapid screening tests such as biosensors [40,41,42,43,44,45] and enzyme-linked immunosorbent assays (ELISA) [46,47,48] are emerging. A further development is their simplification, by developing colored immuno-tests, like rapid disposable membrane-based assay tests or clean-up tandem immune assay column. 

The aim of the current study was to explain how the pH of extraction can lead to misinterpretation of OTA content and the cross-reactivity of CIT on IAC recognizing OTA. We analyze the reason of the underestimation of OTA content in wine after one treatment commonly used to eliminate anthocyans. We compare several methods of extractions and clean-up for OTA and CIT occurrence in wheat samples [4,35,36]. 

## 2. Results and Discussion

### 2.1. Underestimation of OTA Following PEG Clean-Up of Wine

Wine was treated either by poly (1-(2-oxo-1-pyrrolidinylethylene) (PVPP) according to the official method of IOW (International Organization of Wine) or with polyethylene glycol (PEG). PVPP was more efficient than PEG in terms of anthocyanin (respectively 90% and 72%) and polyphenol elimination (respectively 80% and 74%). The amount of OTA after these treatments was measured at 333 nm. Only 16% of OTA was lost after PVPP, whereas the proportion of OTA apparently lost during PEG treatment is over 80%, but simultaneously a new peak appears at 380 nm. When we analyzed the quantity of OTA bound to PEG, we did not find more than 10% of OTA. The effect of PEG concentration and pH on the recovery of OTA from wine using immunoaffinity column clean-up was already investigated by Visconti *et al.* 1999 [49], showing a loss of OTA. We wonder if this underestimation could not be due to an alkalization of the medium, leading to an open ring formation no longer recognized by antibodies, as previously described for breakfast cereals and coffee [50,51,52]. Indeed, the treatment of wine with PEG8000 has shifted from an acidified medium to an alkaline medium (pH recorded was around 9, with a green color), which confirmed the alkalization of the matrix) explaining the formation of the open ring form of OTA (OP-OA) for which the maximum of absorption is around 380 nm [53]. If we measured OTA content using 380 nm, we found 84% of the initial OTA wine content. 

### 2.2. Interference of CIT with OTA on IAC during Analysis of Wheat Samples

#### 2.2.1. Comparison of OTA/CIT Occurrence in Wheat Using Different Methods of Extraction and Clean-Up

A total of 33 wheat samples have been collected either in farms (18 samples) or in wheat cooperatives (15 samples) in northeastern France. OTA and CIT were analyzed either after liquid-liquid extraction in acidic conditions, using an in-house validated method [4] or after purification on the immunoaffinity column (OTA) following the RDT application note [35], or following the official method [36]. In addition CIT was also analyzed using the ELISA test. The analyses were done in triplicate on the same grinded aliquot of wheat. Table 1 shows OTA and CIT contents in these samples using both techniques of clean-up.

Only four samples (F10, F18, S2, S14) were positive for OTA after extraction in alkaline condition and purification onto the immunoaffinity column. The OTA amount ranged from 6.63 ± 2.33 µg/kg to 128.49 + 30.1 µg/kg. Eleven samples (F1, F2, F10, F 15, F18, F19, F20, S2, S9, S14, S16) were positive for OTA after extraction by acetonitrile/water and purification on IAC in PBS pH 7.4, and ranged from 1.00 ± 1.00 to 74.4 ± 8.2 µg/kg. Only three samples (F10, S2, S16) were positive for CIT after ELISA analysis. 

**Table 1:** OTA/CIT content in wheat purified on immunoaffinity column (IAC) [35,36] or by liquid partition [4], expressed in µg/kg (ppb).

Samples	OTA ppb (c)			CIT ppb	
	IAC ^a^/bicarbonate [35]	IAC ^b^/PBS [36]	Liquid-Liquid/acid ^c^ [4]	Liquid-Liquid/acid ^d^ [4]	ELISA ^e^
F1	<LOD	trace	5.58 ± 0.28	<LOD	<LOD
F2	<LOD	trace	6.45 ± 0.33	<LOD	<LOD
F4	<LOD	<LOD	3.14 ± 0.15	<LOD	<LOD
F6	<LOD	<LOD	0.84 ± 0.10	<LOD	<LOD
F7	<LOD	<LOD	0.91 ± 0.11	<LOD	<LOD
F8	<LOD	<LOD	<LOD	<LOD	NA
F9	<LOD	<LOD	Trace (<LOQ)	trace	NA
F10	128.49 ± 30.10	74.40 ± 8.20	6.26 ± 0.31	512.38 ± 61.5	1468.8
F11	<LOD	<LOD	<LOD	0.84 ± 0.10	<LOD
F12	<LOD	<LOD	<LOD	<LOD	NA
F13	<LOD	<LOD	<LOD	<LOD	NA
F14	<LOD	<LOD	1.07 ± 0.06	<LOD	<LOD
F15	<LOD	1.5 ± 1	6.73 ± 0.35	0.86 ± 0.09	<LOD
F16	<LOD	<LOD	<LOD	0.86 ± 0.1	<LOD
F17	<LOD	<LOD	<LOD	0.76 ± 0.1	<LOD
F18	67.23 ± 8.04	67.50 ± 9.50	30.77 ± 1.54	24.57 ± 2.7	NA
F19	<LOD	3.20 ± 0.50	<LOD	7.24 ± 0.58	NA
F20	<LOD	trace	5.20 ± 0.27	<LOD	NA
S2	6.63 ± 2.33	5.07 ± 0.60	1.74 ± 0.09	44.60 ± 4.90	151.5
S3	<LOD	<LOD	<LOD	<LOD	NA
S4	<LOD	<LOD	<LOD	0.86 ± 0.10	NA
S5	<LOD	<LOD	<LOD	<LOD	NA
S6	<LOD	<LOD	<LOD	0.88 ± 0.09	NA
S7	<LOD	<LOD	<LOD	<LOD	NA
S8	<LOD	<LOD	<LOD	1.10 ± 0.09	NA
S9	<LOD	3.06	<LOD	7.46 ± 0.89	<LOD
S10	<LOD	<LOD	<LOD	1.04 ± 0.09	<LOD
S11	<LOD	<LOD	5.3 ± 0.27	<LOD	<LOD
S12	<LOD	<LOD	2.42 ± 0.3	1.57 ± 0.126	<LOD
S13	<LOD	<LOD	<LOD	1.04 ± 0.09	<LOD
S14	27.96 ± 3.01	25.20 ± 2.50	7.59 ± 0.38	3.79 ± 0.30	<LOD
S15	<LOD	<LOD	<LOD	<LOD	NA
S16	<LOD	1.00 ± 1.00	<LOD	15.00 ± 1.80	41.8

^a^ Extraction in alkaline solution [35]; ^b^ extraction in ACN/water [36]; ^c^ LOD OTA = 0.05 µg/kg, LOQ = 0.2 µg/kg; ^d^ LOD CIT = 0.5 µg/kg, LOQ = 1.5 µg/kg; ^e^ LOD CIT (ELISA) = 15µg/kg, NA not analysed.

After acidic extraction and purification by liquid-liquid partition, half of the samples contained OTA ranging from a trace to 30.77 ± 1.54 µg/kg; and CIT ranging from a trace to 512.18 ± 61.5 µg/kg. Samples containing only OTA (F1, F2, F4, F6, F7, F14, F20, S11) were negative after extraction in alkaline condition and purification on IAC. Traces of OTA were observed in the three highest contaminated samples (F1, F2, F20) after extraction with ACN/water and purification on IAC. On the contrary, the samples where OTA was detected in large amount after IAC purification correspond to samples containing simultaneously significant amount of CIT (F10, F18, S2, S14). Three samples apparently positive for OTA after extraction with ACN/water and purification on IAC at pH 7.4 (F19, S9, S16) contained in fact only CIT. This data suggests possible interferences between OTA and CIT for OTA antibodies. 

#### 2.2.2. Recoveries and Confirmation of OTA Metabolites Formed

In order to confirm interference between CIT and OTA, we first pass pure mycotoxins onto IAC. The mycotoxins (OTA and/or CIT) dissolved in ultra-pure water solution or in water solution containing 1% bicarbonate were added in PBS pH 7.4 before IAC. In a second experiment, wheat samples were spiked with OTA alone or in the presence of CIT. One hundred grams of non-contaminated wheat were spiked with OTA at a final concentration equivalent to 100, 40, 7 or 3 µg/kg. Some samples were contaminated with OTA and CIT: (i) 40 µg/kg each toxins (ii) 5 µg/kg OTA ± 50 µg/kg CIT. The samples were analyzed in parallel with the three techniques: acidic extraction followed by liquid-liquid partition purification *versus* alkaline extraction and purification on IAC or ACN/water extraction and purification on IAC. The analyses were made in triplicate. Recoveries are shown in Table 2. 

**Table 2:** Recovery of OTA after classic extraction (acidic condition/liquid-liquid [4]) or IAC (alkaline extraction/IAC purification [35,36]).

		Mycotoxin in aqueous solution			Mycotoxin in wheat	
	IAC/bicarbonate [35]	IAC/PBS [36]	Classic [4]	IAC/bicarbonate [35]	IAC/PBS [36]	Classic
**OTA alone**						
100 µg/kg ^c^	NA	NA	NA	25.80 ± 2.50 (25.8%)	60.10 ± 3.50 (60.1%)	83.50 ± 4.20 (83.5%)
40 µg/kg	17.60 ± 1.40 ^a^ (44%) ^b^	30.40 ± 1.40 ^a^ (66%) ^b^	41.00 ± 1.20 (102%)	11.80 ± 1.87 (29.5%)	24.40 ± 0.87 (61%)	28.80 ± 0.90 (72%)
7 µg/kg	2.03 ± 0.17 (29%)	4.2 ± 0.27 (60%)	6.3 ± 0.18 (90%)	2.24 ± 0.35 (32%)	3.85 ± 0.35 (55%)	5.20 ± 0.16 (74%)
3 µg/kg	<LOD (0%)	1.85 + 0.17 (61.6%)	2.79 ± 0.08 (93%)	<LOD (0%)	1.80 + 0.41 (60%)	2.30 ± 0.07 (76.6%)
**CIT alone**						
5 µg/kg	peak eluting at the OTA retention time	peak eluting at the OTA retention time	4 ± 0.2 (80%)	peak eluting at the OTA retention time	peak eluting at the OTA retention time	3.90 ± 0.12 (78%)
40 µg/kg	peak eluting at the OTA retention time	peak eluting at the OTA retention time	32 ± 1.6 (80%)	peak eluting at the OTA retention time	peak eluting at the OTA retention time	30.00 ± 1.50 (75%)
OTA 40 µg/kg	20.80 ± 3.11 (52%)	62.80 ± 3.11 (157%)	36.00 ± 1.10 (90%)		42.40 ± 0.90 (106%)	28.60 ± 1.43 (71.5%)
CIT 40 µg/kg	-	-	31.20 ± 1.92 (78%)		-	32.00 ± 1.60 (80%)
OTA 5 µg/kg	9.10 ± 0.70 (182%)	7.30 ± 0.70 (146%)	4.50 ± 0.22 (90%)		5.75 ± 0.50 (115%)	3.75 ± 0.11 (75%)
CIT 50 µg/kg	-	-	39.50 ± 3.16 (79%)		-	37.50 + 3.00 (75%)

^a^ Amount of mycotoxin expressed in µg/kg. ^b^ Recovery expressed in percent. ^c^ Recovery for 100 µg/kg in aqueous solution was not tested.

The amount of OTA recovered after IAC following alkaline extraction is less than 50% when the initial OTA amount was in the range of 7 to 40 µg/kg. When the initial concentration of OTA was 3 µg /kg, no more OTA was found after IAC following extraction in alkaline condition. When OTA was spiked on wheat the recovery was of the same order. The amount of OTA recovered after IAC following neutral extraction is better (around 60%) and not dependent of the amount of OTA. In contrast, the recoveries of OTA and CIT after purification by liquid/liquid partition are ranged between 75% and 90%. 

When OTA and CIT were simultaneously passed through IAC either from aqueous solution or from wheat extract, OTA recoveries were much higher than 100%, whereas by liquid/liquid extraction, the recoveries ranged from 75% to 90%. When CIT alone is passed through the IAC, a peak eluting to the same retention time than OTA was detected. We confirm the presence of CIT by mass spectrum analysis (*m*/*z* negative [M − H]^−^ 250 > 232; 217). Thus, CIT is partially recognized by OTA antibodies under neutral and alkaline conditions. 

#### 2.2.3. Effect of the pH on OTA Quantification and UV Spectra in Presence or Absence of CIT

**Figure 3:**
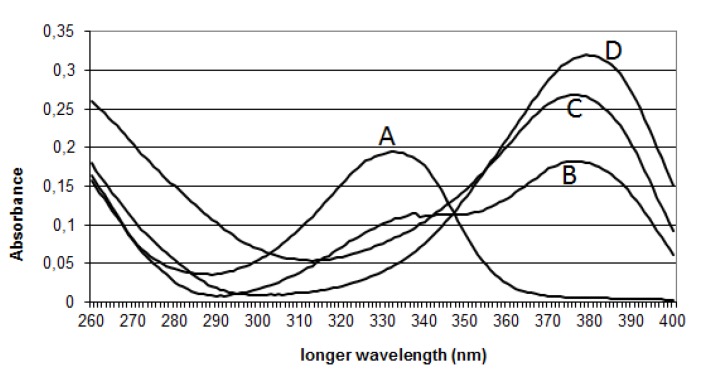
UV-Spectra of ochratoxin A: Curve A represents the spectrum of OTA at pH 4. Curve B represents the spectrum of OTA at pH 7. Curve C represents the spectrum of OTA at pH 8. Curve D represents the spectrum of OTA at pH 12.

**Figure 4:**
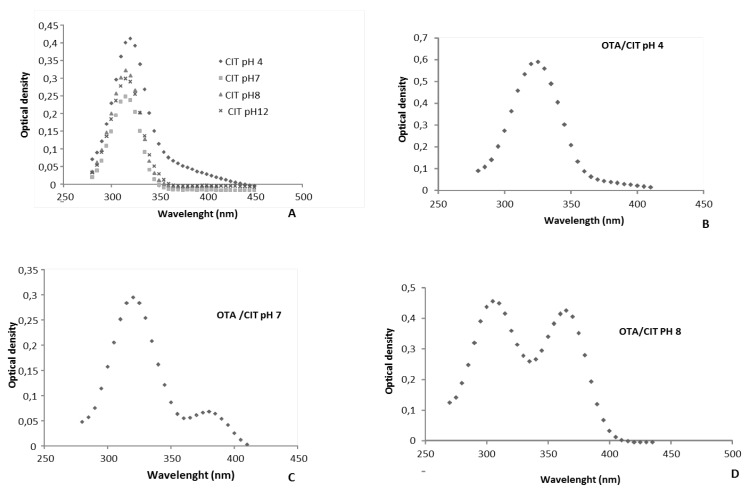
UV-Spectra of citrinin and ochratoxin A in mixture: Curve A represents the spectrum of CIT at four pH (4, 7, 8 and 12). Curve B represents the spectrum of CIT in mixture with OTA at pH 4. Curve C represents the spectrum of CIT in mixture with OTA at pH 7. Curve D represents the spectrum of CIT in mixture with OTA at pH 8.

We recorded the spectra of OTA (Figure 3) or CIT alone at various pH (4, 7, 8 or 12), and in the presence of OTA (Figure 4), in order to understand how the pH of extraction influences OTA or/and CIT analysis.

At pH 4, the spectrum of OTA shows a peak at 333 nm (Figure 3). At pH 7, OTA exists in protonated OTA form corresponding to the peak at 333 nm and in dianion form having a maximal absorbance at 380 nm [50,53,54]. At pH 8, the spectrum shifts to a higher wavelength (around 380 nm) with an increase of the molar absorbance which corresponds to the dianionic form of OTA, and open ring OTA (OP-OA) [50]. At pH 12, the spectrum shifts even to a higher wavelength (around 385 nm) with an increase of optical density, corresponding exclusively to OP-OA. OTA possesses two pKa (pKa = 4.4 (carboxyl group) [54]; pKa = 7.3 (phenol hydroxyl group) [53]). These data confirm those obtained by Verrone *et al.* 2007 [55].

**Figure 5:**
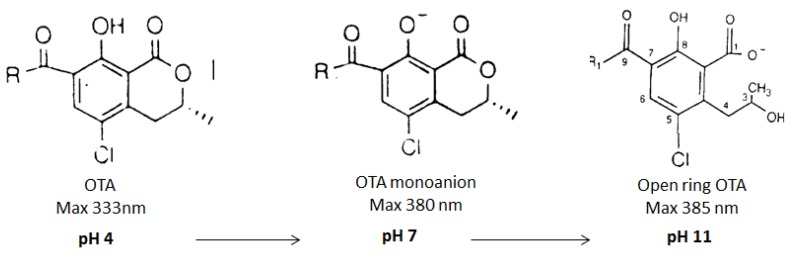
Ring opening of OTA (OP-OA) under alkaline conditions adapted from [50,51,52].

OP-OA is not recognized by antibodies and leads to an underestimated amount. The conversion is reversible. If the pH is lowered, OP-OA is converted into OTA (Figure 5). 

**Figure 6:**
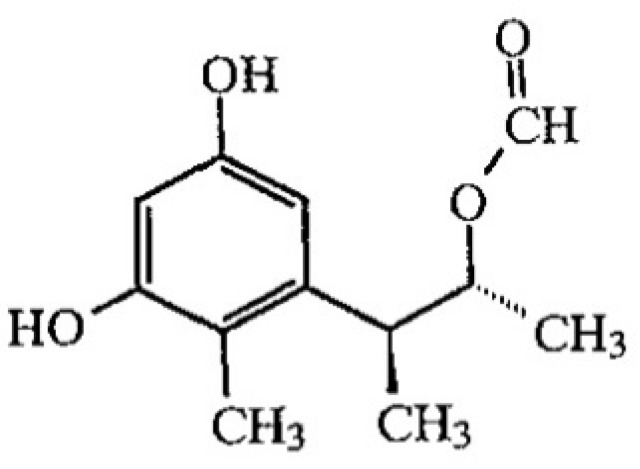
Ring opening of citrinin (citrinin H2) induced by increase of pH [56].

Whatever the pH, CIT presented a peak at 320 nm (Figure 4A), but the intensity of the UV signal is lower at pH > 7 compared to pH 4. The decrease of absorbance could be due to the transformation of citrinin into citrinin H2 (Figure 6) [55] as it was already observed with OTA.

When CIT and OTA are present in the mixture, one or two peaks are observed, depending on the pH (Figure 4B,C,D). At pH 4, the spectrum of the mixture shows only one peak at 320 nm (Figure 4B). At pH > 7 two peaks are observed. At pH 7, the main peak is at 320 nm, and a small peak at 380 nm. At pH > 8, the two peaks have the same intensity (Figure 4D). The appearance of a peak at 380 nm at pH 7 when both toxins are present together can be explained not only by the formation of OP-OA, but also by the formation of ochratoxin quinone (OTHQ) for which the maximum of absorbance is of 380 nm [52]. Chemically, citrinin is a quinone derivative which is pro-oxidant agent susceptible to transform OTA into OTHQ [28]. Simultaneously to the formation of OTHQ, OTA is also converted into OTB (dechlorinated OTA) for which the maximum of absorbance is 318 nm (very close to that of CIT) [52].

The underestimation of the OTA amount under alkaline pH results to the opening of the lactone cycle of OTA (Figure 5), which is no longer recognized by the anti-OTA antibodies [50,51,52]. In presence of CIT, an additional interference occurs with the formation of both OTB and OTHQ that have a similar absorbency to CIT and OP-OA, respectively. OTB is even more so recognized by OTA antibodies [52].

#### 2.2.4. Confirmation by HPLC MS/MS of the Formation of OP-OA; OTHQ and OTB

**Figure 7:**
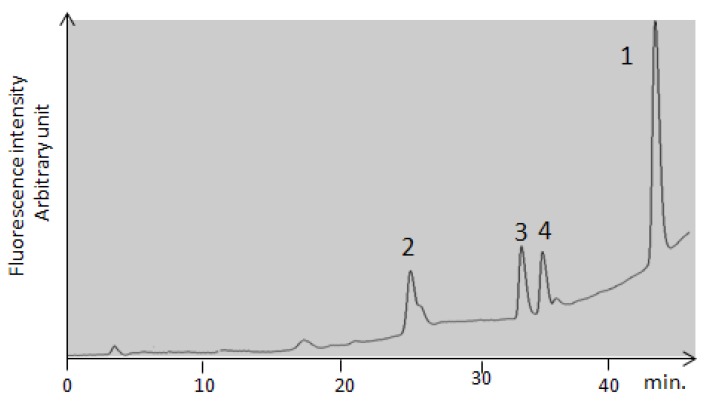
HPLC separation of OTA metabolites from the mixture of OTA and citrinin in aqueous solution.

In order to confirm the hypothesis of oxidation of OTA by CIT, the solution of OTA and the mixtures of OTA and CIT at different pH were analyzed by an HPLC run using the gradient system described by Faucet *et al.* 2006 [57]. An example of the HPLC profile is shown in Figure 7.

Every peak was collected and analyzed by Nano-ESI-IT-MS in negative mode as described by Faucet-Marquis *et al.* 2006 [57]. The spectral data are presented Table 3.

**Table 3:** Spectral data of peak analyzed by nano-ESI-IT-MS.

Peak numbering	Species	RT (min)	λmax	[M−H]^−^	Fragment ions *m*/*z*
1	OTA	42	333	402	358; 314
2	OP-OA	28	380	420	376; 332
3	OTHQ	34	350	384	340; 296
4	OTB	36	316	368	324; 280

The formation of these metabolites depends on the conditions of pH and mixtures (Table 4). 

**Table 4:** OTA metabolites formed at different pH (4, 7, 8, 12) alone and in presence of CIT. -, not detected; +, present; relative intensity, +++ > ++ > +.

peak	1 (OTA)	2 (OP-OA)	3 (OTHQ)	4 (OTB)
OTA alone				
pH 4	+++	-	-	-
pH 7	++	++	-	-
pH 8	+	++	-	-
pH 12	-	+++	-	-
OTA + CIT				
pH 4	+	-	-	-
pH 7		+	+	+
pH 8	+	++	+	+

We confirm that OTA is transformed into OP-OA when the pH increases above 7. The conversion is total when the pH is around 12. At a pH above 7, CIT allows oxidation of OTA inducing formation of OTB (dechlorinated OTA) and OTHQ (quinone derivative). In addition, a part of OTA is transformed into OP-OA.

## 3. Experimental Section

### 3.1. Chemicals

Ochratoxin and citrinin were obtained from Sigma-Aldrich (St Quentin Fallavier, France). All reagents (potassium chloride, sodium hydrogen carbonate, sulfuric acid, phosphoric acid, hydrochloric acid, acetic acid, and sodium dihydrogen phosphate) were of analytical grade. All solvents (methanol, chloroform, acetonitrile, propanol-2, *N*-hexane) were HPLC grade from ICS (Lapeyrousse-Fossat, France).

Ochraprep^®^ and Ridascreen CIT^®^ were obtained from Rhône Diagnostic technologies (RDT) (Saint-Didier au Mont d’or, France).

PEG 8000 (Polyethylene Glycol) and PVPP (polyvinylpolypyrrolidone) were obtained from Promega (Charbonnière, France). 

### 3.2. Preparation Standard Solution

Standard solutions of OTA and CIT were prepared by dissolving 10 mg of OTA or CIT in 1 mL of methanol. Series of working standards from 0.2 to 100 ng/mL of mycotoxin/mL were prepared by dilution in methanol and were used to calibrate the LC detector response. The OTA stock solution was determined by absorbance at 333 nm and calculated with the molar extinction coefficient ε of 5500 mol/cm. CIT stock solution was determined by absorbance at 321 nm and calculated with the molar extinction ε of 5490 mol/cm.

### 3.3. OTA extraction in Red Wine Samples

#### 3.3.1. PEG Treatment

Ten milliliters of wine spiked with OTA (40 µg/mL) were mixed with 10 mL of a solution containing PEG8000 (1%) and NaHCO_3_ (5%). This mixture was incubated during 30 min at room temperature on a rocker. Afterwards, it was centrifuged at 8000 rpm for 15 min.

The spectral analysis (200–800 nm) of the supernatant allows the quantification of the adsorbing effect of PEG for anthocyanins, and the occurrence of polyphenols in wine. 

#### 3.3.2. PVPP Treatment

PVPP, poly(1-(2-oxo-1-pyrrolidinyl)ethylene), is an organic synthetic polymer obtained from the polymerization of *N*-vinyl-2-pyrrolidone. It has the same structure than PVP (pyrrolidinylethylene) but the catalyst used for the polymerization was either sodium hydroxide or *N*,*N*'-divinylimidazolidone. This compound is not soluble in water and has a very high affinity to polyphenols. This characteristic is highly affected by the molecule’s polymerization degree.

According to the IOW (International Organization of Wine), 10 mL of wine (spiked or not with OTA from 5 µg/mL to 20 µg/mL) were diluted with 20 mL of water and supplemented with 10 mg/mL of PVPP. 

### 3.4. Wheat Analysis

#### 3.4.1. Sampling

Thirty-three wheat samples were collected in northeastern France. Eighteen were collected in the farms and 15 in cooperatives.

#### 3.4.2. OTA Extraction from Wheat Samples

OTA was analyzed on wheat samples using two methods of extraction and clean-up: (i) an in-house validated method for the simultaneous extraction/clean-up of OTA and CIT [4]; and (ii) the immunoaffinity column (IAC) [35,36].

##### 3.4.2.1. Solvent Extraction Clean-Up and Partioning Purification

In brief, 20 g of milled sample was acidified with an aqueous solution of potassium chloride (4%) acidified to pH 1.5 with sulfuric acid. The mixture was homogenized and extracted with acetonitrile (ACN). After filtration on Whatman No. 4 paper, the sample is defatted twice with *N*-hexane. The sample was then purified by liquid-liquid partition. 

##### 3.4.2.2. IAC Clean-Up

Two different extractions were made before IAC: one using alkaline extraction, another using a neutral extraction

###### 3.4.2.2.1. OTA Extraction by Bicarbonate

The first method was described in the IAC column (Rhone Diagnostics technologies, Application note Ref No. A9-P14.V1, 1999 [35]). In brief, 10 g of wheat are blended with 200 mL of 1% sodium bicarbonate solution. The sample is filtered on Whatman No. 4 paper. Twenty mL are transferred to the IAC column (3 mL/min). The column is washed with 20% methanol/water (*v*/*v*); flow rate 5 ml/min. OTA is eluted passing slowly 1.5 mL acetic acid/methanol (98/2 *v*/*v*); and after 1.5 mL water. 

###### 3.4.2.2.2. OTA Extraction by Methanol/Water

The second method follows the validated method described by Entwisle *et al.* 2000 [36].

In brief, OTA is extracted from cereals in ACN/water (60/40). The extract is filtered on Whatman No 4 paper. Forty-four mL of PBS pH 7.4 are added to 4 mL of filtrate, and the mixture is transferred onto the IAC. After washing the column with PBS, the OTA is eluted with methanol/acetic acid (98/2) and analyzed by HPLC spectrofluorimetry.

#### 3.4.3. OTA/CIT HPLC Conditions

##### 3.4.3.1. Conditions after Extraction by Liquid/Liquid Extraction [4]

OTA and CIT are analyzed on RP HPLC using C18 column PRONTOSIL 120 (25 cm × 0.46 cm) with inner porosity of 3 µm, under isocratic condition (mobile phase: orthophosphoric acid at 0.33 M/ACN/propan-2-ol (600:400:55), flow rate 0.8 mL/min). Detection is performed with a programmable Merck HITACHI FL Detector L-7485 (excitation 340 nm, emission 465 nm for OTA; 331 and 500 nm for CIT).

##### 3.4.3.2. Condition after IAC Extraction [35,36]

One Hundred µL of extract are injected in HPLC using Spherisorb^®^ ODS2 (25 cm × 0.5 cm) with inner porosity of 5 µm. The mobile phase is ACN/water/acetic acid (51/47/2 *v*/*v*/*v*); flow rate 1 mL/min. Detection was performed with a programmable Merck HITACHI FL Detector L-7485 (excitation 340 nm, emission 465 nm for OTA).

##### 3.4.3.3. Condition for Separation of OTA Metabolites

The metabolites are separated on PRONTOSIL 120 (25 cm × 0.46 cm) with inner porosity of 3 µm, using the following gradient: solvent A: MeOH/ACN/6.5 mM ammonium formate (200/200/600) adjusted to pH 3 with formic acid; solvent B: MeOH/ACN/6.5 mM ammonium formate (350/350/300) adjusted to pH 3 with formic acid. Program: T0 100% A; T10 100% A; T25 30% A; T30 30% A; T45 0% A; T55 0% A; T58. 3.6 CIT extraction and analysis.

CIT was either extracted by liquid-liquid extraction simultaneously with OTA as described above [4] or analyzed using Ridascreen^®^.

## 4. Conclusions

Extraction of OTA in alkaline medium or addition of PEG to wine increasing pH induced an underestimation of OTA content in wheat or wine, respectively. The analyses of OTA/CIT contents in wheat, following purification onto immunoaffinity column (IAC) after alkaline or neutral extraction, led to an overestimation of OTA. This bias is due to the interferences between OTA and CIT for OTA antibodies, but also to the similarity of OTHQ and OTB formed by auto-oxidation of OTA induced by CIT. The pH is crucial for reliable detection of OTA and CIT, and should be below 7 for both accurate liquid/liquid extraction and IAC clean-up. CIT can be partially recognized by OTA antibodies. 
